# Jacksonian Epilepsy

**Published:** 1918-10

**Authors:** 


***Different Forms of Jacksonian Epilepsy in Injuries of the Sulcus Rolandi.*** By H. Meige and A. Benisty. *Société de Neurologie de Paris*, Jan. 1918.

Jacksonian epilepsy of classic type is the one observed most of all after injuries of the sulcus rolandi. The most usual form, motor, would be better denominated sensitivo-motor, the sensations being as frequent as the motor disturbances. It is to be noted that Jacksonian phenomena appear in regions affected with motor or sensory disturbances.

Jacksonian crises have almost never been seen without brachial or facial monoplegia in men suffering from genuine cortical paraplegia. This might be only a coincidence. Jacksonian phenomena, however, seem to have been very frequent when a well-marked facial monoplegia has been accompanied by a hemiplegia or a brachial monoplegia. In certain cases, mostly hemiplegia, the affection extends to the arm and even to the inferior limb. Difficulty of speech and constriction of the throat precede the loss of consciousness in cases where the facial monoplegia is very well marked, and when the Jacksonian wave extends from the arm to the face. This seems to be the regular type in cases having a conjugated deviation of the head and eyes after the injury, and when the facial monoplegia and speech disorders have been severe at the onset. Some cases, between the attacks, have convulsion of certain limbs without numbness, electrization, dyspnea, speech disturbances, or loss of consciousness. In the intervals between the crises, certain motor disorders are frequently seen, and their nature seems to be very likely coinitial. They are involuntary movements occurring in attacks during the intervals between the crises, and localized to a limb affected with Jacksonian disturbances. Also tremors of a limb or even of half of the body, occurring a few days before the epileptic crisis, and caused by emotion or fatigue, are sometimes observed. All those intermittent motor disorders can be considered as abortive attacks.

The authors draw attention to another group of accidents which are not so well-known: the continuous movements in head wounds, with or without coinitial phenomena (a. Rapidly oscillating tremors, mostly at the extremity of limbs, increasing with effort or fatigue, and decreasing at rest. b. Rapid clonic movements. c. Choreiform movements: tendons slowly contracted, imitating the slow movements of chorea). The occurrence of those continuous movements in cranium injury naturally suggests a permanent cortical irritation. These are similar to cases in which we see, immediately after the injury and before the onset of the paralysis, limbs which will be paralysed later animated with clonic tremors, accompanied by dyspnea, disorders of speech and loss of consciousness. Similar facts are also noted before or after an ictus. The tremor, or the pre- or post-hemiplegia hemichorea are classical; and we must not be surprised to see them occurring in brain injuries.

Jacksonian paresia is a little known form of Jacksonianism. (*Société Médicale des Hôpitaux*, Oct., '17). The attacks are sudden and transitory, occurring in one limb only (side opposite to injury); in both limbs; or, very seldom, in the limb on the same side. Vaso-motor troubles are constant in all the cases that have been observed. At the moment of the crisis the limbs become cold and asphyxiated. This suggests that the concomitant motor and vaso-motor disorders are due to an intercranial sympathetic pertubation. The Jacksonian phenomena were long ago attributed to circulatory disturbances. If the location of the regulating center of circulation in the encephalum is still unknown, the existence of this center appears to be plausible. The Jacksonian equivalent represented by the motor and vasomotor paresia confirms this theory.

